# Thriving at work as a mediator of the relationship between psychological resilience and the work performance of clinical nurses

**DOI:** 10.1186/s12912-024-01705-6

**Published:** 2024-03-22

**Authors:** Zhou-Min Shen, Yang-Yang Wang, Yi-Min Cai, Ai-Qun Li, Yu-Xin Zhang, Hong-Jiao Chen, Yuan-Yuan Jiang, Juan Tan

**Affiliations:** 1 Department of Nursing, Hunan Provincial People’s Hospital, The First Affiliated Hospital of Hunan Normal University, Changsha, 410005 People’s Republic of China; 2 Department of Nursing, Hunan Normal University, Changsha, 410013 People’s Republic of China; 3 Department of Nursing, The Second Xiangya Hospital of Central South University, Changsha, 410012 People’s Republic of China

**Keywords:** Thriving at work, Resilience, Work performance, Nursing management

## Abstract

**Objective:**

This study aims to investigate the relationship between psychological resilience, thriving at work, and work performance among nurses, as well as analyse the mediating role of thriving at work in the relationship between psychological resilience and the work performance of nurses. The findings are intended to serve as a reference for nursing managers to design tailored work performance intervention programs.

**Method:**

Using convenience sampling, 308 clinical nurses were selected from a tertiary hospital in Changsha City, Hunan Province, China, from February to April 2023. The Connor–Davidson Resilience Scale (CD-RISC), the Thriving at Work Scale, and the Work Performance Scale were employed for the questionnaire survey. Pearson correlation analysis was used to explore the relationship between psychological resilience, thriving at work and work performance. The SPSS 26.0 software’s ‘Process’ plugin was utilised for mediation effect analysis.

**Results:**

Significantly positive correlations were found between psychological resilience and thriving at work (*r* = 0.806, *P* < 0.01), thriving at work and work performance (*r* = 0.571, *P* < 0.01) as well as psychological resilience and work performance (*r* = 0.572, *P* < 0.01). Psychological resilience significantly predicted work performance positively (*β* = 0.558, *t* = 11.165, *P* < 0.01), and this prediction remained significant when thriving at work (the mediating variable), was introduced (*β* = 0.371, *t* = 4.772, *P* < 0.01). Psychological resilience significantly predicted thriving at work positively (*β* = 0.731, *t* = 20.779, *P* < 0.01), and thriving at work significantly predicted work performance positively (*β* = 0.256, *t* = 3.105, *P* < 0.05). The mediating effect size of thriving at work between psychological resilience and work performance was 33.49% (*P* < 0.05).

**Conclusion:**

Thriving at work plays a partial mediating role between psychological resilience and work performance. The level of work performance among clinical nurses was relatively high. Nursing managers can enhance thriving at work by fostering psychological resilience among clinical nurses, thereby further improving their work performance to ensure high-quality and efficient nursing care.

## Background

As medical reforms continue to progress and the general public’s demand for health services increases, there is an urgent societal need for the expansion and enhanced comprehension of both the intrinsic and extrinsic dimensions of nursing services. Natural disasters have occurred frequently in recent years, and nurses have become the largest rescue force in disaster emergencies. Nurses play a vital role in disaster prevention, mitigation and recovery. However, disaster response nurses may experience psychological problems such as acute stress disorder and post-traumatic stress disorder during or after rescue operations [1]. Work performance is the tangible application of clinical nurses’ acquired knowledge and skills, a measure of their efficiency and effectiveness in achieving set goals, and an embodiment of individual capabilities in the work environment resulting in contributions and accomplishments. Yildirim believes that nurses’ work performance is sensitive to various work-related conditions [2]. However, the current high workload and frequent sudden incidents in China’s clinical settings can lead to various psychological burdens for clinical nurses, causing emotional and physical fatigue [3] and affecting the quality and efficiency of nursing work [4].

Psychological resilience, also known as psychological elasticity, is a positive adaptive capability displayed by an individual when facing adversity and can be divided into three components: tenacity, strength and optimism [5]. Resilience is essential for frontline health workers to cope with adverse situations, especially during public health emergencies [6]. Good psychological resilience can help nurses respond swiftly, enhance their ability to cope with work pressure, prevent work fatigue and potential psychological problems and is associated with positive coping strategies, which can help to elevate their work performance [7]. Thriving at work, also known as vigour at work, is a state of feeling active and energetic at work, reflecting an individual’s work enthusiasm [8]. A study based on the conservation of resources theory found that a proactive personality and positive emotions among employees can promote work prosperity [9]. Studies have also confirmed that psychological resilience has a certain predictive effect on thriving at work [10], raising nurses’ thriving at work level while reducing work burnout. Research indicates that individuals often shift their emotions, behaviours and attitudes from one domain to another [11]. This suggests that nurses with weak psychological resilience often bring their anxieties about their lives outside of work or about family into their clinical work, leading to job burnout or making mistakes easily. While scholars at home and abroad have shown considerable interest in the concept of thriving at work, there is a paucity of studies investigating this concept within the realm of nursing.

The current research generally focuses on evaluating the level of psychological resilience of nurses and exploring its influencing factors [12]. Factors of the medical work environment (e.g. excessive workload, emotional stress related to the work of dying patients, problems with patients and family members, night shift work, conflicts with managers) reduced the performance and life satisfaction of both doctors and nurses [13]. A predictive correlational study presented a novel framework that emotional intelligence (EI) is vital for effective work performance among nurses, and EI was found to be a useful coping strategy against occupational stress [14]. Moreover, there are currently no documented studies on the complex relationships among clinical nurses’ psychological resilience, thriving at work and work performance.

To achieve positive medical outcomes, the administrators of medical institutions must first identify factors and potential mechanisms related to psychological resilience, thriving at work and the work performance resilience of front-line healthcare workers (especially the large group of nurses). Therefore, this study aims to evaluate and examine how nurses’ psychological resilience and thriving at work influence work performance. Additionally, the study explores the correlations among these three variables. The results aim to provide a reference for nursing managers to design tailored work performance intervention programmes.

### Theoretical and conceptual framework

**Fig. 1:**
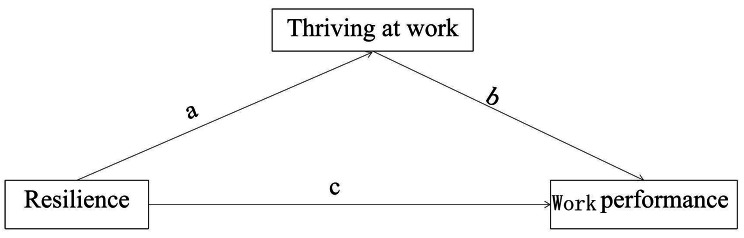
The hypothesis diagram

The contingency theory of work and performance was chosen as the conceptual framework for guiding this study. This theory was proposed by Boyatzis in 1982 as a response to the failure of traditional management methods to maximise employee performance. The theory views effective performance as a dependent variable and points to three main foundations of effective performance, that is, individual, work-related and organisational environmental factors, and posits that consistency between two or more components can improve performance efficiency [15]. The psychological resilience explored in this study is a relevant correlational factor at the individual level that fully reflects the influence of individual subjective initiative on work performance. We developed a conceptual research model based on variables from the contingency theory of work and performance (Fig. 1).

Consistent with the conceptual framework that guides our study, the following hypotheses were empirically tested:

Hypothesis a: Nurses’ psychological resilience significantly predicts their ability to thrive at work.

Hypothesis b: Nurses thriving at work significantly predicts their work performance.

Hypothesis c: Nurses’ psychological resilience significantly predicts their ability to thrive at work.

Hypothesis d: Nurses’ thriving at work plays a partial mediating role between psychological resilience and work performance.

## Methods

### Research participants

A convenience sampling method was used to select clinical front-line nurses from a tertiary A-level hospital in Changsha, Hunan Province, China, from February to April 2023 as the research participants for the survey. Inclusion criteria: (1) possession of a nurse practising certificate; (2) clinical working time ≥ 1 year. Exclusion criteria: (1) intern nurses, continuing education nurses; (2) executive grade nurses, nurses assigned full time for tasks other than general nursing care, such as at reception and information desk; (4) nurses who were not in the hospital during the survey due to sickness or personal long-term leave; (3) past or current sufferers of mental disorders.

This study obtained ethical approval from the hospital’s ethics committee (ethical approval no: [2023]-144) and informed consent from the research participants.

### Sample size calculation

The sample size required for this study is based on the results calculated by G*Power 3.1™. Statistical power was 95%, α = 0.05 and the effect size was moderate (F2 = 0.15); two predictors were used as the statistical basis for calculating linear multiple regression analysis (fixed model, R^2^ deviation 0). According to the calculation, the sample size should be 262. In addition to a 10% system bias and sample loss rate, the total sample size should be at least 289. Finally, 308 samples were confirmed for inclusion.

### Data collection

Survey research was conducted using an electronic questionnaire compiled through the Wenjuanxing platform (wjx.cn). The questionnaire included the research survey’s purpose, content and methods of completion. The researchers contacted the head nurses of each ward and issued the questionnaire QR code via WeChat, following a set of standardised instructions, for the clinical nurses to complete anonymously. To ensure the quality of the responses, all items were set as mandatory fields that required completion to submit the form. The data was exported from the user backend of Questionnaire Star, which is a practical app that can design questionnaires, distribute online and collect information. A total of 308 questionnaires were collected, 298 of which were valid, resulting in a valid recovery rate of 97%.

### Research methods

#### Research tools

(1) General Information Questionnaire: A self-designed survey form was used, which included gender, age, length of service, education, marital status, whether they were an only child, details about children, type of employment, job title, position, current department, average number of night shifts per month, average total monthly income (including salary, bonuses, night shift fees and various benefits) and whether they enjoyed nursing work.

(2) Psychological Resilience Scale (Connor–Davidson Resilience Scale [CD-RISC]): This scale was compiled by Connor and Davidson in 2003 and translated and revised into Chinese by Yu X et al. [16]; it is widely used among various populations in China. The Chinese version of the CD-RISC consists of 25 items covering three dimensions: tenacity, strength and optimism. It uses a Likert 5-point scoring method, with items rated according to the frequency of the described situation, from 0 (never) to 4 (always), with a total score from 0 to 100. The higher the score, the stronger the individual’s adaptability and the higher the level of psychological resilience. A score of ≥ 65 indicates good psychological resilience. In this study, the overall Cronbach’s α coefficient for the scale was 0.846, while the three subscales’ Cronbach’s α coefficients were 0.947, 0.935 and 0.812.

(3) Thriving at Work Scale: This scale was compiled by Porath et al. [8] in 2012 and translated into Chinese and revised by Deng Jiaxin [17]. The scale contains a total of 8 items, encompassing 2 dimensions: learning and vitality. The scale uses a Likert 5-point scoring method, with each item rated according to the described situation from 1 (strongly disagree) to 5 (strongly agree) for a total score of 8 to 40. The higher the score, the higher the nurse’s thriving at work. In this study, the overall Cronbach’s α coefficient for the scale was 0.796, and the two subscales’ Cronbach’s α coefficients were 0.898 and 0.895, respectively.

(4) Work Performance Scale: A work performance survey form, developed by Van Scotter and Motowidlo, was utilised, which was translated into Chinese and revised by scholars in China [18]. The scale contains 11 items encompassing three dimensions: task performance, interpersonal facilitation and job dedication. The scale uses a Likert 5-point scoring system, with a total score of 55. The higher the score, the higher the individual’s job performance. In this study, the overall Cronbach’s α coefficient for the scale was 0.815, and the three subscales’ Cronbach’s α coefficients were 0.849, 0.910 and 0.801.

### Statistical methods

The SPSS 26.0 software was used for statistical analysis. An independent samples t-test or one-way analysis of variance was used for group comparisons. For normally distributed measurement data, mean ± standard deviation ($\stackrel{-}{x}\pm s$) was used and for count data, frequency and percentage were used. Pearson correlation analysis was used to explore the relationship between psychological resilience, thriving at work, and work performance. The ‘Process’ plugin in the SPSS software was used for mediation effect analysis. All statistical significance was set at *P*<0.05.

To test the hypotheses of this study, we adopted the method of Baron and Kenny [19], which proposes four steps of regression analysis to determine that independent variables influence dependent variables through mediating variables. This method first regresses the independent variable with the mediator, then the mediator with the dependent variable and then the independent variable with the dependent variable to confirm that there is a significant zero-order association between the variables [19]. When the significant zero-order correlation between variables was assumed, step 4 was carried out. As such, step 4 required simultaneous multiple linear regression, including predictors, mediators and dependent variables. When indirect effects were present but no direct effects, we inferred a complete mediation; conversely, when both direct and indirect effects were present, we confirmed partial mediation [20].

## Results

### Clinical nurses’ work performance scores

The total score for the work performance of clinical nurses was 44.72 ± 6.10. The mean scores for each dimension, ranked from high to low, were as follows: interpersonal facilitation, task performance and job dedication. The results of the one-way analysis showed statistically significant differences in the work performance scores of clinical nurses based on age, length of service, type of employment, job title, position, average number of night shifts per month and whether they enjoyed nursing work (*P* < 0.05). See Table 1 for details.

**Table 1:** Univariate analysis of clinical nurses’ work performance (*n* = 298, $\overline x \pm s$)

Item	Number	Percentage (%)	Work Performance Score	t/F-value	***P***
Gender				-1.367	＞0.05
Female	273	91.6	44.58 ± 5.96		
Male	25	8.4	46.32 ± 7.45		
Age (years)				12.038	＜0.01
18–29	112	37.6	43.37 ± 6.39		
30–39	142	47.7	44.63 ± 5.61		
≥ 40	44	14.8	48.5 ± 5.44		
Years of Service (years)				8.831	＜0.01
1–3	46	15.4	42.54 ± 6.58		
4–7	72	24.2	43.22 ± 5.98		
8–15	123	41.3	45.02 ± 5.72		
≥ 16	57	19.1	47.75 ± 5.47		
Education				0.287	＞0.05
Diploma and below	68	22.8	44.91 ± 6.31		
Bachelor and above	230	77.2	44.67 ± 6.06		
Employment Type				3.368	＜0.01
Regular Employment	154	51.7	45.86 ± 5.96		
Contract/Personnel Agency	144	48.3	43.51 ± 6.05		
Professional Title				8.610	＜0.01
Nurse	54	18.1	43.02 ± 6.67		
Senior Nurse	105	35.2	43.47 ± 5.92		
Nurse-in-charge	126	42.3	45.94 ± 5.60		
Deputy Chief Nurse and above	13	4.4	50.15 ± 4.54		
Position				8.595	＜0.01
Staff Nurse	222	74.5	43.96 ± 6.40		
Charge Nurse/Head Preceptor	38	12.8	45.82 ± 4.68		
Head Nurse	38	12.8	48.11 ± 3.97		
Current Department				2.024	＞0.05
Internal Medicine	81	27.2	43.83 ± 5.78		
Surgery	64	21.5	45.42 ± 5.96		
Obstetrics and Gynecology	45	15.1	46.27 ± 5.91		
Pediatrics	32	10.7	42.97 ± 7.19		
Critical Care	35	11.7	43.91 ± 6.35		
Outpatient	15	5.0	47.53 ± 5.91		
Other	26	8.7	44.77 ± 5.38		
Average Number of Night Shifts per Month (time)				8.152	＜0.01
0	82	27.5	47.43 ± 5.48		
1–4	124	41.6	43.94 ± 6.20		
5–9	85	28.5	43.45 ± 5.93		
≥ 10	7	2.3	42.43 ± 4.04		
Like Nursing Work or Not				4.44	＜0.01
Yes	205	68.8	45.75 ± 5.86		
No	93	31.2	42.46 ± 6.06		

### Common method bias test

The Harman single-factor test was used to check for common method bias. The results showed that there were 6 factors with eigenvalues greater than 1, and the maximum factor variance explanation rate was 48.63%, which is lower than the critical value of 50%, suggesting that there was no serious common method bias.

### Correlation analysis of clinical nurses’ psychological resilience, thriving at work and work performance

The results showed a significant positive correlation between psychological resilience and thriving at work (*r* = 0.806, *P* < 0.01), between thriving at work and work performance (*r* = 0.571, *P* < 0.01) and between psychological resilience and work performance (*r* = 0.572, *P* < 0.01). See Table 2 for details.

**Table 2:** Correlation analysis of clinical nurses’ psychological resilience, thriving at work, and work performance (*n* = 298, *r*-value)

Dimension	Psychological Resilience	Thriving at work	Work Performance
Psychological Resilience	1.000	-	-
Thriving at work	0.806^a^	1.000	-
Work Performance	0.572^a^	0.571^a^	1.000

Note: ^a^*P*＜0.01

### Analysis of the mediating effect of thriving at work on the relationship between psychological resilience and work performance among clinical nurses

**Fig. 2:**
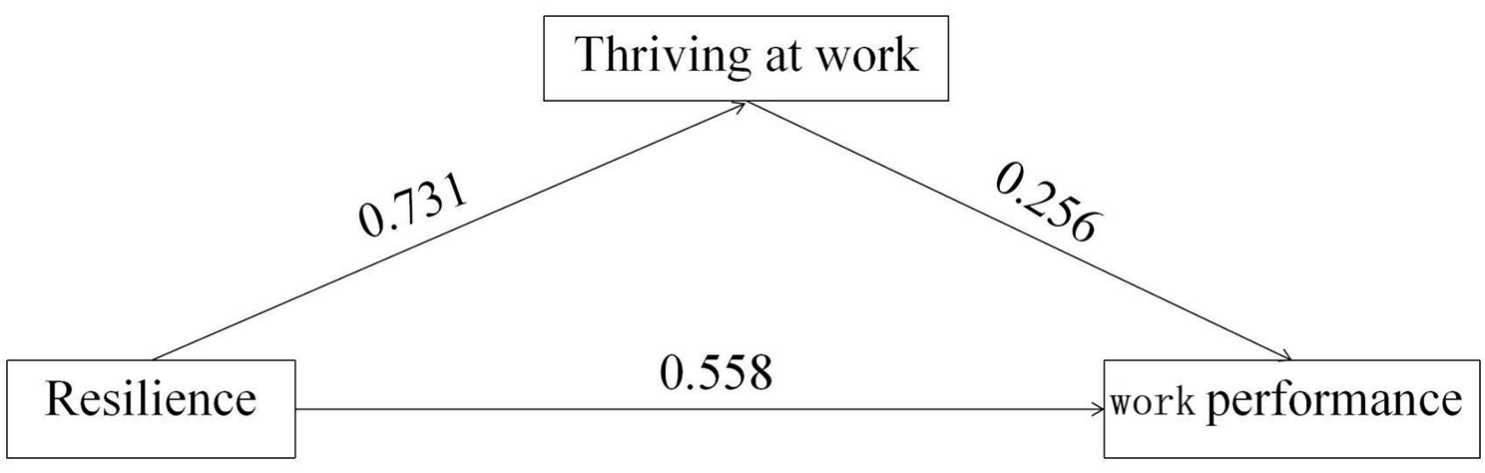
The validation diagram

To further analyse the impact of psychological resilience on work performance, the mediating effect of thriving at work on work performance was tested. Model 4 in the Process macro (a plug-in of SPSS software) was employed for the mediation effect analysis, with age, length of service, type of employment, job title, position, average number of night shifts per month, years of work, and whether they enjoyed nursing work as control variables. The bootstrap method was used for the confidence interval (CI) estimation test, with 5,000 resamples, to calculate the 95% CI. The results indicated that psychological resilience had a significant positive predictive effect on work performance (*β* = 0.558, *t* = 11.165, *P* < 0.01). Even after incorporating thriving at work as a mediating variable, the predictive effect of psychological resilience on work performance remained significant (*β* = 0.371, *t* = 4.772, *P* < 0.01). Psychological resilience had a significant positive predictive effect on thriving at work (*β* = 0.731, *t* = 20.779, *P* < 0.01), and thriving at work had a significant positive predictive effect on work performance (*β* = 0.256, *t* = 3.105, *P* < 0.05). The mediating effect of thriving at work between psychological resilience and work performance was 33.49%, and the direct effect of psychological resilience on work performance was 66.51%. The bootstrap 95% CI of the direct effect of psychological resilience on work performance and the mediating effect of thriving at work did not include 0 in the upper nor lower limits, indicating that the differences were statistically significant (*P* < 0.05). See Tables 3 and 4 for details and Fig. 2 for the validation diagram.

**Table 3:** Analysis of mediating effects of thriving at work on the relationship between psychological resilience and work performance among clinical nurses (*n* = 298)

Outcome Variable	Predictive Variable	R	R^2^	F(df)	B	***t***-value	***P***
Work Performance		0.631	0.398	23.838^a^			
	Age				0.071	0.769	＞0.05
	Years of Service				0.097	0.980	＞0.05
	Employment Type				-0.049	-0.870	＞0.05
	Professional Title				0.058	0.747	＞0.05
	Position				0.104	1.944	＞0.05
	Average Number of Night Shifts per Month				-0.027	-0.486	＞0.05
	Like Nursing Work or Not				-0.013	-0.264	＞0.05
	Psychological Resilience				0.558	11.165	＜0.01
Thriving at work		0.838	0.702	85.100^a^			
	Age				-0.034	-0.521	＞0.05
	Years of Service				0.080	1.153	＞0.05
	Employment Type				0.005	0.129	＞0.05
	Professional Title				-0.074	-1.351	＞0.05
	Position				0.048	1.281	＞0.05
	Average Number of Night Shifts per Month				-0.112	-2.860	＜0.05
	Like Nursing Work or Not				0.169	4.746	＜0.01
	Psychological Resilience				0.731	20.779	＜0.01
Work Performance		0.646	0.417	22.894^a^			
	Age				0.079	0.875	＞0.05
	Years of Service				0.076	0.782	＞0.05
	Employment Type				-0.050	-0.906	＞0.05
	Professional Title				0.077	1.001	＞0.05
	Position				0.092	1.734	＞0.05
	Average Number of Night Shifts per Month				0.002	0.028	＞0.05
	Like Nursing Work or Not				-0.057	-1.093	＞0.05
	Psychological Resilience				0.371	4.772	＜0.01
	Thriving at work				0.256	3.105	＜0.05

Note: ^a^*P*＜0.01

**Table 4:** Decomposition of mediating effects

Effects	Effect Value	SE	95%CI	Relative Effects (%)
Total Effect	0.228	0.018	0.191, 0.262	
Direct Effect	0.152	0.031	0.090, 0.210	66.51%
Indirect Effect	0.076	0.023	0.032, 0.122	33.49%

## Discussion

### Current status of clinical nurses’ work performance

The results of this study indicated that the average work performance score of clinical nurses was 4.07 ± 0.56, indicating a high level of work performance. This result is consistent with the findings of Liu Lingyan et al. [21], suggesting that most clinical nurses feel good about their performance at work and believe they are competent in their clinical roles. Among these, the interpersonal facilitation dimension scored higher than the other two dimensions, indicating that clinical nurses feel good about their handling of interpersonal relationships. Moreover, this study found that the work performance of clinical nurses was slightly lower than that of general practitioners [22] and hospital infection control professionals [23], which may be related to the high intensity of clinical nursing work, high pressure, low income, irregular rest and lower social status of the role. In addition, clinical nurses, apart from fulfilling their own job responsibilities, also need to complete the nursing department’s regular three basics and three strict exams, which can easily lead to job burnout, which is unfavourable for clinical nursing work and learning. This suggests that medical and health institutions and nursing managers, based on local conditions, should improve the bonus distribution and appraisal systems, optimise incentive mechanisms and further promote the work enthusiasm of clinical nurses to enhance their work performance.

This study also found that age, length of service, type of employment, job title, position, average number of night shifts per month and whether they enjoyed nursing work were factors that influenced work performance, and this was consistent with previous research findings [24–28]. The reasons for these findings are analysed as follows. (1) With an increase in age and length of service, nurses gain a deeper understanding of professional knowledge, accumulate more work experience and enhance their stress resilience and problem-solving abilities. This enables them to complete clinical nursing work at a higher quality in the shortest time, ultimately improving their work performance. (2) Officially employed nurses have higher work performance, which may be associated with the current welfare treatment in public institutions in China. (3) The higher the job title and position, the higher the income of clinical nurses. Additionally, clinical nurses with lower titles and no positions were typically less experienced, had been working for a lower number of years and were incapable of handling high-difficulty nursing tasks. They were often assigned to simple and trivial chores, which could easily reduce individual occupational identity and self-efficacy [29, 30], lead to lower work enthusiasm and further affect work performance. (4) Clinical nurses who frequently work night shifts may face problems concerning energy and time allocation conflicts. Coupled with the high stress of night shifts, even if they want to efficiently complete their work and studies, they often have the will but not the energy, leading to lower work performance. (5) Compared with clinical nurses who dislike nursing work, those who enjoy it tend to have a higher sense of professional identity. They can face nursing work with a positive attitude, tend to have a high level of work initiative and show a willingness to invest more enthusiasm and energy in their work, thereby improving work performance.

### Correlation between psychological resilience, thriving at work, and work performance in clinical nurses

Psychological resilience for individual protection is an important mechanism through which one can demonstrate flourishing positive work behaviours [31]. This study found a positive correlation between psychological resilience and work performance, that is, the higher the level of psychological resilience, the better the work performance, consistent with the research results of Liu Ruihan et al. [32]. Due to the shortage of nursing staff and the complexity of their work, nurses are often overloaded. Good psychological resilience helps individuals generate positive emotions, which enables them to better complete nursing tasks, manage relationships with colleagues and easily pass periodic hospital assessments. Thus, it enhances work performance concerning three different levels: task performance, interpersonal promotion and work dedication. This study also found that psychological resilience correlates positively with thriving at work, that is, the higher the level of psychological resilience, the stronger the thriving at work, which is in line with previous research findings [10]. This suggests that it is necessary to emphasise the perspective of positive psychology and pay attention to the motivational aspects of the work when evaluating the work performance of clinical nurses. Research in organisational psychology describes thriving at work as a subjective state of consciousness in which an individual becomes so absorbed in something that they forget about time, fatigue and everything else [33]. When clinical nurses face external stressors and other stimuli, psychological resilience serves as a characteristic factor that helps them to overcome adversities and develop positively. It promotes individuals to be passionate and energetic about work and learning in terms of three aspects: optimism, strength and resilience, thereby increasing thriving at work. Additionally, this study reveals a positive correlation between thriving at work and work performance, that is, the stronger individuals thrive at work, the better the work performance, which echoes previous findings [34]. This implies that clinical nurses with strong thriving at work can devote more energy to learning the skills and methods needed for their tasks, adapt to dynamic changes in the work environment, and improve work efficiency, leading to better work performance.

### The mediating role of thriving at work between psychological resilience and work performance

The survey results revealed that thriving at work partially mediates the relationship between psychological resilience and work performance. Specifically, clinical nurses’ psychological resilience positively influences work performance. Furthermore, clinical nurses’ psychological resilience can also indirectly affect work performance through thriving at work, with the mediating effect accounting for 33.49% of the total effect. The Socially Embedded Model of Thriving at Work [35] postulates that stable organisational situational characteristics and resources generated in dynamic work will together trigger proactive individual behaviours, leading to the development of thriving at work. Flexible psychological adjustment is a powerful motivator for improving job engagement and has also been shown to have a beneficial effect on job satisfaction and is a strong predictor of job engagement [36]. Clinical nurses in good psychological health will possess psychological traits such as optimism, resilience and self-confidence, which will aid them in comfortably acquiring new professional knowledge and skills, effectively dealing with stressful events, maintaining amicable colleague relationships and navigating complex nurse–patient interactions. This enhances their professional skills and generates stable organisational situations, fostering a higher level of thriving at work. Their work and learning become more active, ultimately contributing to personal growth and career development.

## Practical implications and recommendations

Our research filled the knowledge gap concerning the relationship between the psychological factors and work performance of clinical nursing staff and explored a variety of factors affecting work performance. The results of this study should be carefully considered by leaders and healthcare policymakers to develop systematic strategic plans to reduce the stressors in the nursing work environment, improve the psychological resilience of nurses and avoid the negative effects of exogenous and endogenous factors on their work performance. Therefore, it is suggested that nursing managers should pay close attention to individual differences, focus on the intrinsic needs of clinical nurses in their career development process and emphasise alleviating unnecessary work pressure on clinical nurses through, for example, flexible scheduling, professional title promotion and offering official employment to enhance their occupational identity and, consequently, elevate work performance.

On the one hand, nursing managers should pay attention to the psychological needs of clinical nurses when developing personalised training programmes. This can be achieved by cultivating a positive departmental atmosphere through mutual respect between superiors and subordinates and colleague support, giving nurses a sense of security and belonging. Proactive interventions to improve the psychological resilience of clinical nurses should also be implemented, thereby enhancing their thriving at work, and infusing vitality and enthusiasm into their work. On the other hand, the needs of clinical nurses for off-site continuing education and learning should be thoroughly considered. Opportunities for off-site learning should be provided, helping clinical nurses obtain support, resources and information; moreover, learning and growth opportunities through activities such as off-site continuing education and specialised training courses should also be made available. This will enable them to continually accumulate medical knowledge and professional skills in their daily nursing work, thereby improving work performance and patient satisfaction.

## Limitations

This study has certain limitations. The study was conducted in a medical institution in Changsha. The convenience sampling method may not fully represent other nurses in different regions and institutions. Second, because our data was collected through self-reporting, we cannot ensure the honesty of the respondents. As a result, the possibility of bias is increased. It is necessary to adopt different sampling methods and larger samples for further research to strengthen the current research findings and better understand the factors affecting the work performance of nursing workers in China’s tertiary hospitals. Future studies can focus on psychological resilience, thriving at work and the work performance of clinical nurses in different regions and hospitals of different levels. Furthermore, qualitative and longitudinal research can be strengthened to provide guidance and references for nursing managers in formulating reasonable intervention plans for improving clinical nurses’ work performance.

## Conclusion

In this study, the work performance level of clinical nurses was relatively high, and thriving at work played a partial mediating role between the psychological resilience and work performance of clinical nurses. This suggests that, while considering the quality of nursing care and patient satisfaction, nursing managers should also emphasise cultivating psychological resilience and thriving at work among clinical nurses. This will help to promote enthusiasm for learning and commitment to clinical work, thereby improving nurses’ work performance.

## Data Availability

The datasets used and/or analysed during the current study available from the corresponding author on reasonable request.
